# Morbidity and Mortality Associated With Respiratory Virus Infections in Allogeneic Hematopoietic Cell Transplant: Too Little Defense or Harmful Immunity?

**DOI:** 10.3389/fmicb.2018.02795

**Published:** 2018-11-21

**Authors:** Anne Birgitta Versluys, Jaap Jan Boelens

**Affiliations:** ^1^Blood and Marrow Transplantation Program, Princess Maxima Center for Pediatric Oncology, Utrecht, Netherlands; ^2^Stem Cell Transplant and Cellular Therapies Program, Pediatrics, Memorial Sloan Kettering Cancer Center, New York, NY, United States

**Keywords:** respiratory virus infection, allogeneic transplant of hematopoietic stem cells, alloimmunity and transplantation, lung disease, outcome

## Abstract

The impact on morbidity and mortality of Community Acquired Respiratory Virus (CARV) infections in patients undergoing Allogeneic Hematopoietic Cell Transplant (HCT) is widely studied. Here we give an overview of the current literature on the incidence and chance of progression to severe disease in this highly immune compromised population. We discuss the issue whether it is predominantly direct viral damage that causes clinical deterioration, or that it is in fact the allogeneic immuneresponse to the virus that is most important. This is an important question as it will guide therapeutic decision making. It asks for further collaborative studies focusing on sensitive surveillance with PCR techniques and relating clinical data with parameters of immune reconstitution.

## Introduction

Community acquired respiratory virus infections (CARV) include a variety of viruses such as rhinovirus, coronavirus, respiratory syncytial virus (RSV), influenza virus, Para influenza virus, and metapneumo virus. CARV infections range from asymptomatic carriership to significant respiratory disease. Prevalence of CARV largely depends on season, detection mode, age of patient and immune status (Shah et al., 2012; Hirsch et al., 2013; Green, 2017). Influenza and RSV have significant seasonal variation, whereas Para influenza or rhinovirus cause disease year round (Green, 2017). Highly sensitive diagnostic techniques like polymerase chain reaction (PCR) for the detection of viral DNA and RNA reveal a high prevalence of CARV in the normal population. In healthy children attending day care prevalence is as high as 40% (Moe et al., 2016). In children admitted to hospital for respiratory disease 43–75% are tested PCR positive, almost twice as high as in adults admitted to hospital for respiratory disease (Ching et al., 2018). In the immune compromised pediatric population the prevalence of CARV is around 50–80% (Fazekas et al., 2012) with mostly mild symptoms at time of detection.

There are many reports on CARV prior to, or early after, Hematopoietic Cell Transplantation (HCT). Recently a large multicenter retrospective analysis in 1,560 pediatric HCT recipients showed an incidence of 16.6% symptomatic CARV infections within 1 year after transplant (Fisher et al., 2017). Surveillance studies in the same population on Nasopharyngeal Aspirates (NPA) routinely performed prior to transplant showed an incidence of 50% (Versluys et al., 2010).

Most studies discuss the risk of progression to viral pneumonia, for various types of CARV. Only few groups looked at long term outcome. Results are conflicting, reported risk of progression is 5–75% (Bredius et al., 2004; Hirsch et al., 2013; Chemaly et al., 2014b; Fisher et al., 2017; Green, 2017). HCT is a curative treatment for several malignant and non-malignant childhood diseases. Its success is limited by infections, allo-immunity and toxic events. Respiratory viruses contribute to post-transplantation morbidity and mortality in different ways.

Here we provide an overview of recent literature on CARV in the HCT setting, focusing on the risk of progressive viral lung disease, the role of viruses in the lung microbiome and the potential viral trigger for allo-immunity after HCT.

## Community acquired respiratory viruses and HCT

Reported incidence of **RSV** in HCT-recipients varies between 1 and 17% (Shah et al., 2012; Robinson et al., 2015; Fisher et al., 2017), depending largely on season, patients' age and detection methods. Progression to LRTI occurs in 18–55% (Shah et al., 2012; Kim et al., 2014; Fisher et al., 2017), with risk factors for progression related to age, donor source, use of steroids, immune status, and concomitant infections (Renaud et al., 2013; Chemaly et al., 2014b; Kim et al., 2014; Shah et al., 2014). Mortality rates are around 10% (Chemaly et al., 2014a; Robinson et al., 2015; Fisher et al., 2017) It is important to notice the in most pediatric studies, RSV-positive patients were treated with the antiviral drug ribavirin (Molinos-Quintana et al., 2013), with anti-RSV monoclonal antibodies (palivizumab) or with non-specific intravenous immunoglobulins (IVIG) (El-Bietar et al., 2016), or with a combination (Chávez-Bueno et al., 2007). Aerosolized ribavirin and IVIG is recommended for adult HCT recipients with RSV LRTI (Dignan et al., 2016; Waghmare et al., 2016).

**Para influenza virus (PIV)** in HCT recipients is systematically reviewed by Shah et al. (2016a). The incidence of PIV in HCT recipients is 5% (range 0.2–19%), 36% progressing to LRTI. Significant predictors of LRTI progression were infection within 100 days after HCT, lymphocytopenia/neutropenia at the onset of infection, use of corticosteroids, younger age, and respiratory co-infections. Reported overall mortality is 10% (0–37%), in PIV-LRTI 27%. There is currently no licensed therapy for PIV pneumonia.

**Influenza** is diagnosed in approximately 1–5% of HCT recipients, and in up to 33% of patients with respiratory symptoms in the flu-season. Progression to viral pneumonia occurs in 14–30% of patients, and is associated with mortality in 10–28% (Fisher et al., 2017; Green, 2017). These numbers are strongly influenced by seasonal outbreaks and the subtype of the influenza virus. Risk factors for progressive influenza disease are lymphocytpenia/neutropenia and steroid use (Kmeid et al., 2016). In contrast to many other respiratory viruses, influenza can be treated with neuraminidase inhibitors (Waghmare et al., 2016; Green, 2017).

**Human rhino virus** (hRV)is the most common cause of respiratory virus infections in both immunocompetent and immunocompromised individuals. Reported incidence in HCT recipients is 8–36% (Versluys et al., 2010; Shah et al., 2012; Campbell et al., 2015; Fisher et al., 2017). For a long time there was uncertainty about the ability of hRV to cause lower respiratory tract disease. Recent studies however, suggest that hRV may be a clinically significant pathogen with the potential to cause serious pulmonary disease in HCT recipients (Campbell et al., 2015; Seo et al., 2015, 2017; Versluys et al., 2017) with risk of progression to LRTI of 9–24% (Shah et al., 2012; Campbell et al., 2015; Fisher et al., 2017) and hRV related mortality of 4–33% (Shah et al., 2012; Campbell et al., 2015; Fisher et al., 2017).

There is increasing interest in the pathogenecity of **human metapneumo virus (hMPV)**. In a systematic review (Shah et al., 2016b) Shah et al. summarized all published data on hMPV in patients with hematologic malignancies or undergoing HCT. About one third of described cases were children. They report an overall incidence of 5% (range 0–40%), with a risk for progression to LRTI of 34% (range 0–100%) and a mortality rate of 6% (0–17%) in the total hMPV positive group, and 27% in the hMPV-LRTI group.

Other respiratory viruses like **bocavirus (BoV)** and **coronavirus (CoV)** in HCT setting, are scarcely studied. Prevalence of either BoV or CoV is 26% in the adult HCT population with respiratory symptoms. A significant proportion of the CoV infected patients required hospitalization, and some progressed to LRTI. In contrast, BoV detection was rare and almost always related to co-pathogens (Pinana et al., 2018). A surveillance study on BoV in children found 10% of children with RTI to be PCR positive, as well as 17% of the healthy controls; thus showing a high prevalence of the virus without necessarily causing disease. Progression to lower infection however was associated with higher BoV load and viremia, suggesting a pathogenic role in a subgroup of patients (Christensen et al., 2010).

**Figure 1:**
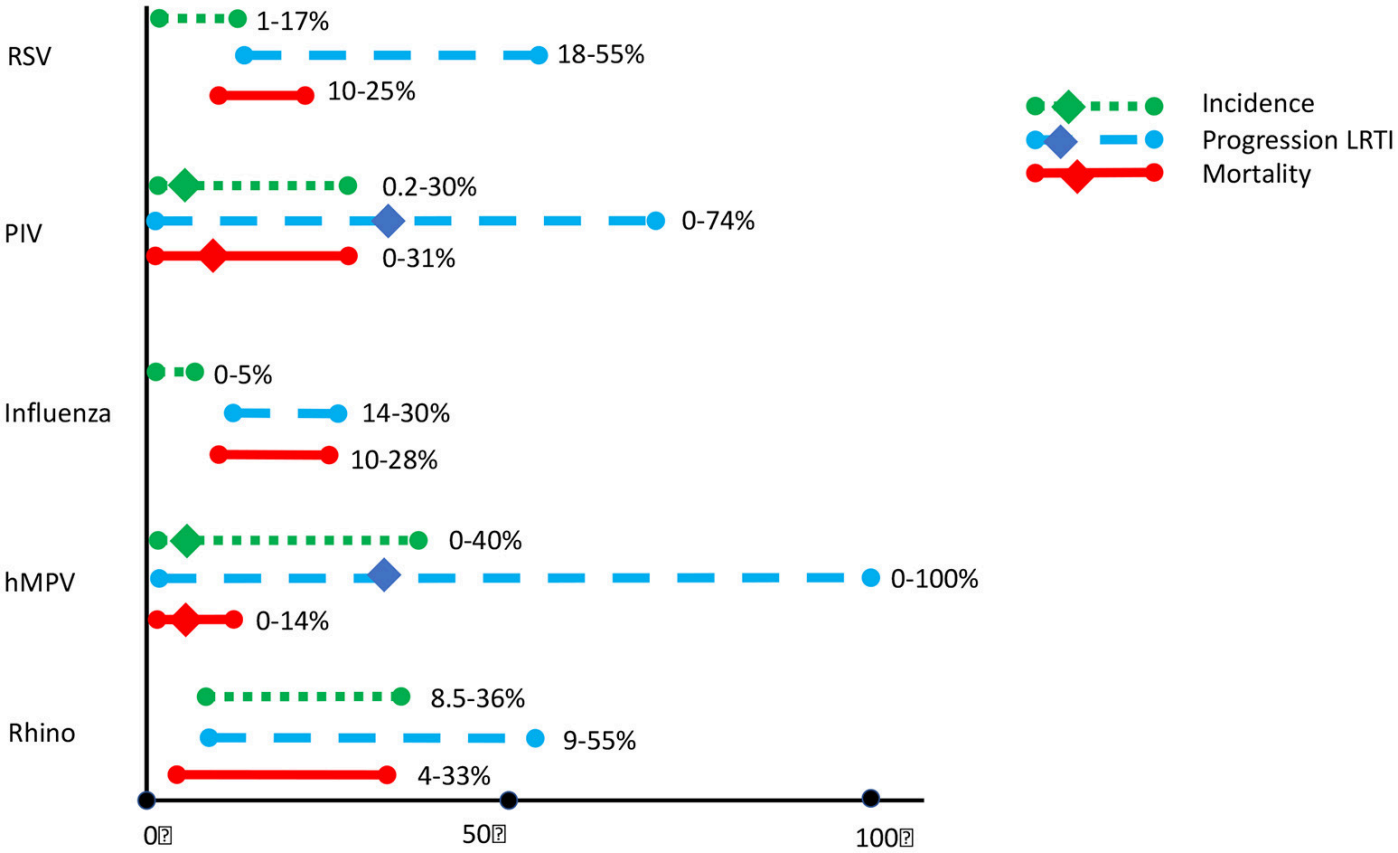
Incidence of common respiratory viral infections (CRV) and associated progression to Lower Respiratory Tract Infections (LRTI) and mortality in HCT recipients. Updated from Shah et al. (2012), based on (Versluys et al., 2010; Shah et al., 2012; Chemaly et al., 2014b; Campbell et al., 2015; Robinson et al., 2015; Fisher et al., 2017; Green, 2017).

Figure 1 shows the incidence of CARV in mainly adult HCT population, and the impact of CARV on LRTI and mortality. Profound immunosuppression in patients undergoing HCT obviously leads to a greater risk of infection, with prolonged shedding of the virus, a higher chance of transmission of disease and a greater risk of progression to severe lower respiratory tract disease. As antiviral therapy is only effective in the minority of the respiratory viruses, and vaccines are not widely available, prevention is very important.

The working group of the fourth European conference on infections in leukemia (ECIL-4) reviewed the literature on CARV in leukemic patients and patients after HCT (Hirsch et al., 2013). For prevention they recommend infection control measures like good personal hygiene, avoiding contact with individuals with a respiratory tract infection and restricting young children from visiting patients. Administration if IVIG preparations in patients with hypogammaglobulinemia (IgG < 4 g/l), and the use of intravenous monoclonal antibody specific for the RSF-F protein (palivizumab) during RSV outbreaks may be considered. They do not advocate routine screening for CARV. Deferral of chemotherapy or conditioning should be considered, as well as treatment for RSV and HPIV only in the HCT setting.

In 2016 a joint working group in the UK (Dignan et al., 2016) has reviewed the available literature and made recommendations for the diagnosis and management of respiratory viral infections in patients with hematological malignancies or those undergoing hematopoietic stem cell transplantation. As far as prevention is concerned they come to the same conclusions as ECIL-4, and add the recommendation for influenza vaccinations in household contacts and medical staff, and post-exposure prophylaxis with oseltamivir in HCT patients who have been in contact with influenza.

## Respiratory viruses after HCT and clinical outcome

In a recent large prospective study, including 458 adults and children undergoing allogeneic HCT, clinical outcomes associated with respiratory viruses (RV) detected prior to HCT were analyzed (Campbell et al., 2015). Multiplex PCR testing for RV was done on nasal washes or nasopharyngeal swabs. In 25% of patients a RV was detected, 22% of them being asymptomatic. In the pediatric subgroup, defined as aged < 18 years, the prevalence of RV was higher (46%), with a larger proportion being without symptoms (37%). The RV positive patients were significantly younger and had higher risk underlying disease with lower lymphocyte count. Overall mortality at day 100 was significantly higher in RV-patients than in non-RV patients (15.5 vs. 7.9%; HR 2.4; 95% CI 1.3–4.5; *p* = 0.07). In 27% of the deceased patients the cause of death was thought to be directly related to the pre-HCT RV, no data are given about the cause of death in the other patients. Patients with Rhinovirus performed worse compared to the other RV. This may be partly explained by the fact that most patients with RSV or influenza where treated with antiviral therapy or had their transplant delayed. Data on longer follow up are lacking (Campbell et al., 2015).

Hutspardol et al. retrospectively studied treatment related mortality (TRM) and long-term pulmonary complications in 32 children who had respiratory symptoms and a RV detected within 100 days after allogeneic HCT. The overall frequency of documented RV infections was 6.5%, half of the patients presented with signs of a LRTI and mortality rate at day 100 was 13%. Cause of death was pneumonitis/ARDS in all, with symptoms occurring on day 11–98 after HCT. During follow up (4.3 years, range 1.4–11.8) no chronic pulmonary complications nor allo-immune lung syndrome was observed (Hutspardol et al., 2015).

With regard to long term pulmonary function Chien et al. studied 1,130 adult HCT recipients by performing routine pulmonary function tests years after HCT. Airflow obstruction, defined as an annualized decline in FEV1 of more than 5%, occurred in 26% of patients and had impact on overall mortality. Higher age at transplant, GVHD category, pulmonary function pre-transplant and the occurrence of a respiratory virus infection within the first 100 days after HCT were significant risk factors for airflow obstruction (Chien et al., 2003). Erard et al. further studied the association of RV and airflow decline, and found that this was particularly true in patients after LRTI caused by parainfluenza virus or respiratory syncytial virus (Erard et al., 2006).

In a retrospective study among 1,560 pediatric HCT recipients in 9 US centers 16.6% acquired symptomatic RV within the first year after HCT (Fisher et al., 2017). In line with others, rhinovirus was the most common virus, followed by RSV and PIV. RV was detected after a median of 56 (11–151) days after HCT. Most children had URTI only, in patients with hMPV there was significantly more LRTI. During 3 months follow up 15% required mechanical ventilation and 14% had significant pulmonary sequelae like bronchiolitis obliterans, subacute pulmonary problems and other not specified pulmonary complications. All cause mortality among RV positive patients was 11%, compared to 5% in the non-CARV group. Recent steroid exposure and RV detection within 60 days after HCT were poor prognostic factors for morbidity and death. At least 50% of death were not attributable to CARV infection. The timing of the events is also remarkable, as 61% of deaths occurred more than 30 days after diagnosing CARV infection, which is at least 3 months after HCT for most.

The widespread use of PCR diagnostics has led to an increase in the detection of CARV in patients undergoing HCT. Many of these patients become symptomatic and a significant proportion develops LRTI. There is a clear increased risk for mortality in CARV positive patients. Hence, prevention and development of anti-viral drugs are of great importance.

However, one could debate about the reason for severe morbidity and mortality in CARV positive patients. How do you diagnose progressive viral infection? The CARV will not be cleared for months because of the immunocompromised state of the host after HCT, so finding positive PCRs is not convincing enough. Timing of (progression of) symptoms in relation to immune reconstitution might be helpful in answering the question if it is progression of viral damage or if the donor derived immunity actually is targeting the lung.

## Respiratory viruses (RV) and alloimmunity

In last decade more and more evidence has emerged that “triggered” alloreactivity may play a crucial role in toxicity and mortality. This holds true for HCT, but is also recognized in solid organ transplantation. In the context of lung transplantation several studies have examined the role of RV in the development of chronic lung allograft dysfunction (CLAD), a form of chronic rejection of the lung (Kumar et al., 2005, 2010; Fisher et al., 2016). Many, but not all, reported an association between RV and CLAD. Pooled analyses of studies on RV and CLAD (Vu et al., 2011) did not confirm the association, mainly due to the heterogeneity of studies and limitations in design, diagnostic techniques and definitions. Fisher et al. tried to overcome these limitations by studying a more homogenous cohort of lung transplant recipients, using modern molecular assays to detect RV and applying consensus definitions of CLAD (Fisher et al., 2016). In 250 patients, 50 (20%) developed CLAD at a median of 95 weeks (interquartile range (IQR): 53–157 weeks). In 79 patients (31.6%) a respiratory viral episode was seen, after a median of 19 weeks post lung transplantation (IQR: 9–63 weeks). In multivariate analysis RV was associated with CLAD (HR1.9, 95% CI 1.1–3.5; *p* = 0.03). This association was stronger the more proximate the RV occurred after lung transplantation.

Our group studied the role of respiratory viruses (RV) in immune mediated lung disease after HCT, analogous to this phenomenon as described after lung transplantation (Versluys et al., 2010, 2017). The host-vs.- graft chronic allograft rejection in lung transplantation is in many ways comparable to the graft-vs.- host inflammation in hematopoietic cell transplantation.

In a cohort of 179 children undergoing allogeneic HCT routine NPA and BAL sampling for the presence of RV was done prior to transplant. RV was found in 61% (41% in BAL/NPA, 20% in NPA-only). Rhinovirus was the most frequently detected RV (42%). Allo-immune lung syndrome (Allo-LS), defined as Bronchiolitis Obliterans Syndrome (BOS) or Idiopathic Pneumonia Syndrome (IPS), occurred in 13%, after a median of 8.5 weeks (1–28 weeks). RV-positivity in BAL was a predictor for Allo-LS (HR 3.8, 95% CI 1.4–10.7; *p* = 0.01). No other predictors were found. The hypothesis is that RV causes epithelial damage and triggers an allogeneic immune response leading to severe lung disease. So the lung disease does not occur primarily from progressive viral infection during the period of low immunity, but from allo-immune mediated damage weeks after HCT.

This inflammatory aspect of disease might explain the reported benefit of steroid use on the risk of mechanical ventilation among HCT recipients with influenza (Choi et al., 2011), and the controversy on risks of steroid use in case of CARV after HCT (Waghmare et al., 2016).

## Viruses and the respiratory microbiome

More and more is known about the role of microbiota in human health. So far research has largely focused on the gut microbiome, also in the context of GvHD. But the microbial ecosystem at other body sites, including the respiratory tract is attracting growing attention. Host and environmental factors influencing the respiratory microbiota include genetics, microbial exposure (birth mode, feeding type, day care), vaccination, infections and antibiotics (Man et al., 2017). Viral infection interacts with the microbiome by disrupting the airway epithelial barrier facilitating bacterial adhesion, liberating host derived nutrients and decreasing muco-ciliary clearance. In addition respiratory viruses can modulate innate and adaptive immune responses promoting bacterial colonization (Man et al., 2017). Moreover, it is becoming clear that the virome should be seen as a part of the microbiome, that affects the function of the host immune system (Cadwell, 2015). The role of a disturbed respiratory microbiome/virome in lung disease is postulated for asthma and chronic obstructive pulmonary disease (COPD) (Zou et al., 2016).

## Discussion

Impact of CARV on outcomes after HCT is an intriguing topic where pathogenesis is not completely understood. Is the poor immune system associated with progressive infection? Does allo-immunity play a crucial role in lung toxicity?

Most studies describe data on symptomatic patients where CARV is detected at time of symptoms. Only few studies report on pre-HCT sampling, although we know a large proportion of our patients is CARV positive with only mild symptoms. Time of (worsening) of symptoms, warranting viral diagnostics and thus detecting the CARV, is often weeks after HCT. Are these nosocomial acquired viruses, or were these virus already present and giving symptoms after a certain period of time? Are the viruses acquired after discharge? But then immunity is usually restored to a certain degree.

From various infectious diseases, like RSV bronchiolitis (Fonseca et al., 2018), immune reconstitution inflammatory syndrome (IRIS) in HIV patients (with cryptococcal meningitis, CMV retinitis or BCG-itis) (Walker et al., 2015) or IRIS in non-HIV immunesuppressed patients with immune recovery (followed by worsening of treated tuberculosis, idiopatic pneumonia or hepatitis) (Sueki et al., 2018), we know the harmful effect of immune response on the patient. The debate about inappropriate immune response on infectious triggers is especially intriguing in the allogeneic setting.

The definition criteria for alloimmune mediated lung syndromes (Panoskaltsis-Mortari et al., 2011; Jagasia et al., 2015) describe the clinical, radiologic and functional aspects of lung pathology, with exclusion of other evident causes of this phenotype, like heart failure and infection, including respiratory virus infection. One can argue if this holds true for respiratory viruses detected by PCR. The detection modes have become much more sensitive over time, so the impact of positive findings on the disease criteria should be reevaluated. In the HCT population with its high prevalence of RV, these viruses will have a long persistence making them detectable for weeks after initial infection, with uncertain meaning for their role in pathology.

An interesting paper in this matter was recently published by Seo et al. (2015). In 69 patients with IPS, they went back to BAL samples at time of diagnosis, and applied more sensitive diagnostics for microbial pathogens. In 56% of patients an occult pathogen was found, 36% being a respiratory virus. All patients were treated with steroids because of IPS. Overall mortality was higher in the group of patients with an occult pathogen, than in the group without. The authors conclude that these patients had had to be excluded as IPS patients, that they had infectious pneumonia and that steroid treatment had adversely influenced their outcome.

However, as we are not informed about RV status pre-HCT, this could also be persisting RV after HCT, triggering immune-mediated lung disease (IPS). In that situation steroids are beneficial in the treatment at the moment of clinical deterioration.

In conclusion, despite the growing awareness of CARV infections in HCT patients, well-designed studies are lacking that systematically evaluate diagnostic and therapeutic strategies of CARV. Only then we will be able to better understand the direct viral impact and the indirect alloimmune pathology, both largely influencing clinical outcome of patients.

Detailed longitudinal studies, combining data from microbioma/virioma surveillance with data on immunerecovery after HCT and clinical outcome are needed to better understand pathogeneic mechanisms involved in lung disease and CARV after HCT. This insight should largely influence the therapeutic decision of delaying transplant, treating RV and most important increasing or decreasing immune suppression after transplant.

## Author contributions

All authors listed have made a substantial, direct and intellectual contribution to the work, and approved it for publication.

### Conflict of interest statement

The reviewer PV declared a past co-authorship with one of the authors JB to the handling editor. The remaining author declares that the research was conducted in the absence of any commercial or financial relationships that could be construed as a potential conflict of interest.
